# Supplementary figures and images for: Three types of remapping with linear decoders: A population-geometric perspective
Source: PLoS Comput Biol. 2025 Oct 3;21(10):e1013545. doi: 10.1371/journal.pcbi.1013545 (PMC12510668; doi:10.1371/journal.pcbi.1013545)

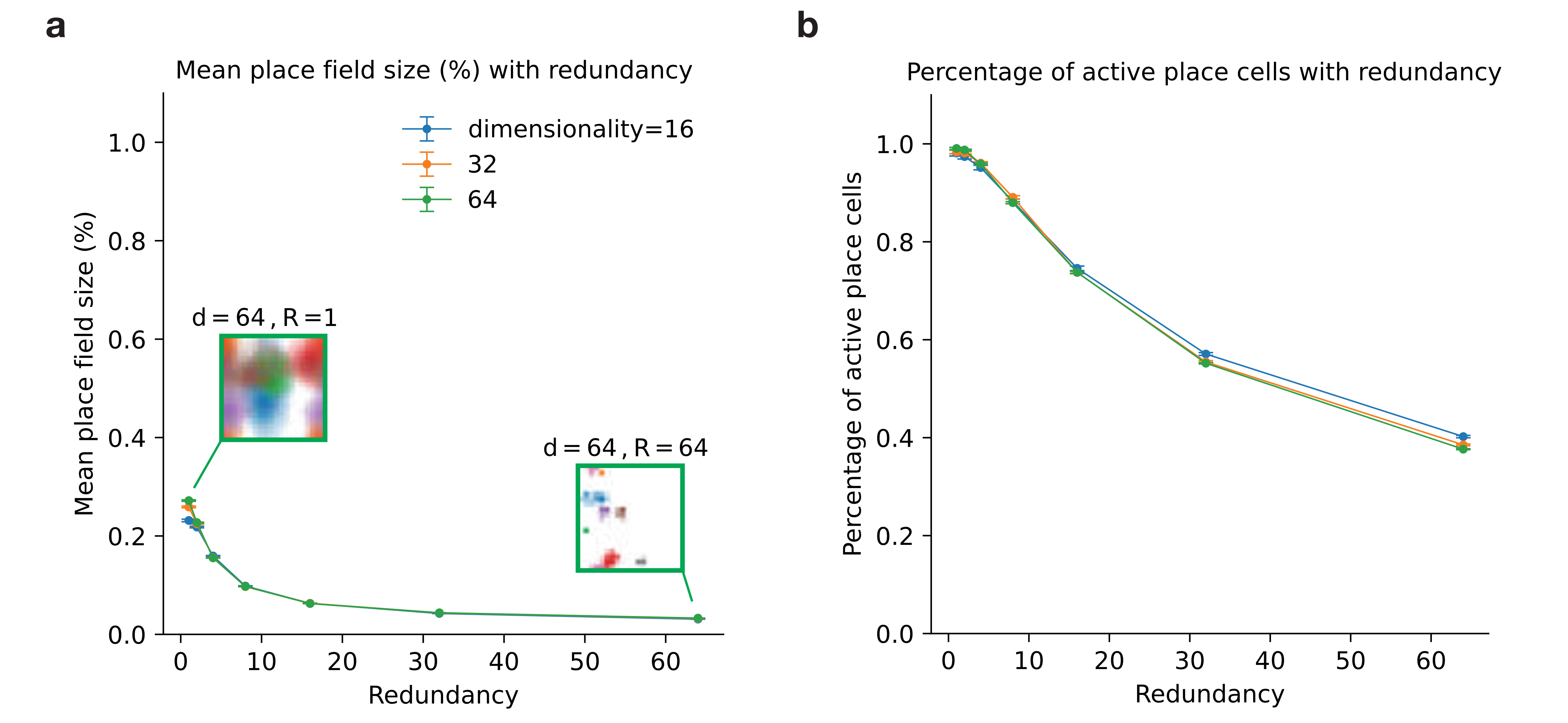

Supplement: S1 Fig — a,b: Mean place field size (as % of the environment; a) and percentage of active place cells in each environment ( b) as a function of redundancy (N/Y) for three different dimensionalities (Y) for multi-chart ED (mean across neurons and 10 environments, with SEM across environments). Example place fields shown in ( a) plotted for (Y,redundancy)=(64,1) and (64,64). Related to the distribution of percentage of neurons active in n rooms (see S3c Fig). (TIFF) [file pcbi.1013545.s001.tiff]

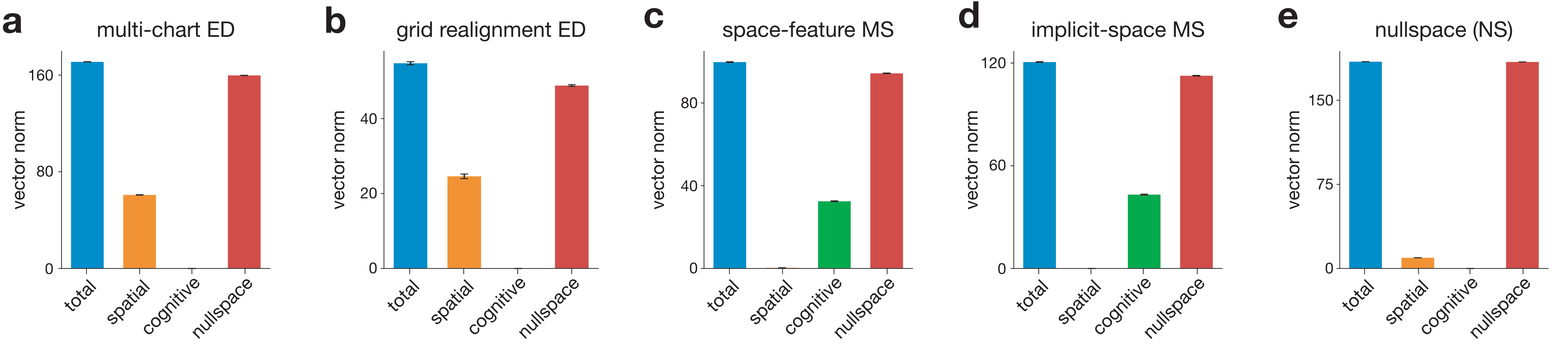

Supplement: S2 Fig — Norm of the remapping vector between 10 environments computed as total (||𝐫A−𝐫B||); spatial (||𝐃𝐩𝐫A−𝐃𝐩𝐫B||, which is equal to ||𝐲A−𝐲B||=||(𝐑A−𝐑B)𝐳𝐩|| for ED and NS remapping and ||𝐳𝐩A−𝐳𝐩B|| for MS remapping); cognitive (||𝐃𝐜𝐫A−𝐃𝐜𝐫B||=||𝐳𝐜A−𝐳𝐜B||); and NS (||(𝐫A−𝐫B)−𝐄((𝐳A−𝐳B)||=||νA−νB||). Shown for main figure examples of multi-chart ED remapping ( a), grid realignment ED remapping ( b), space-feature MS remapping ( c), implicit-space MS remapping ( d), and NS remapping ( e). (TIFF) [file pcbi.1013545.s002.tiff]

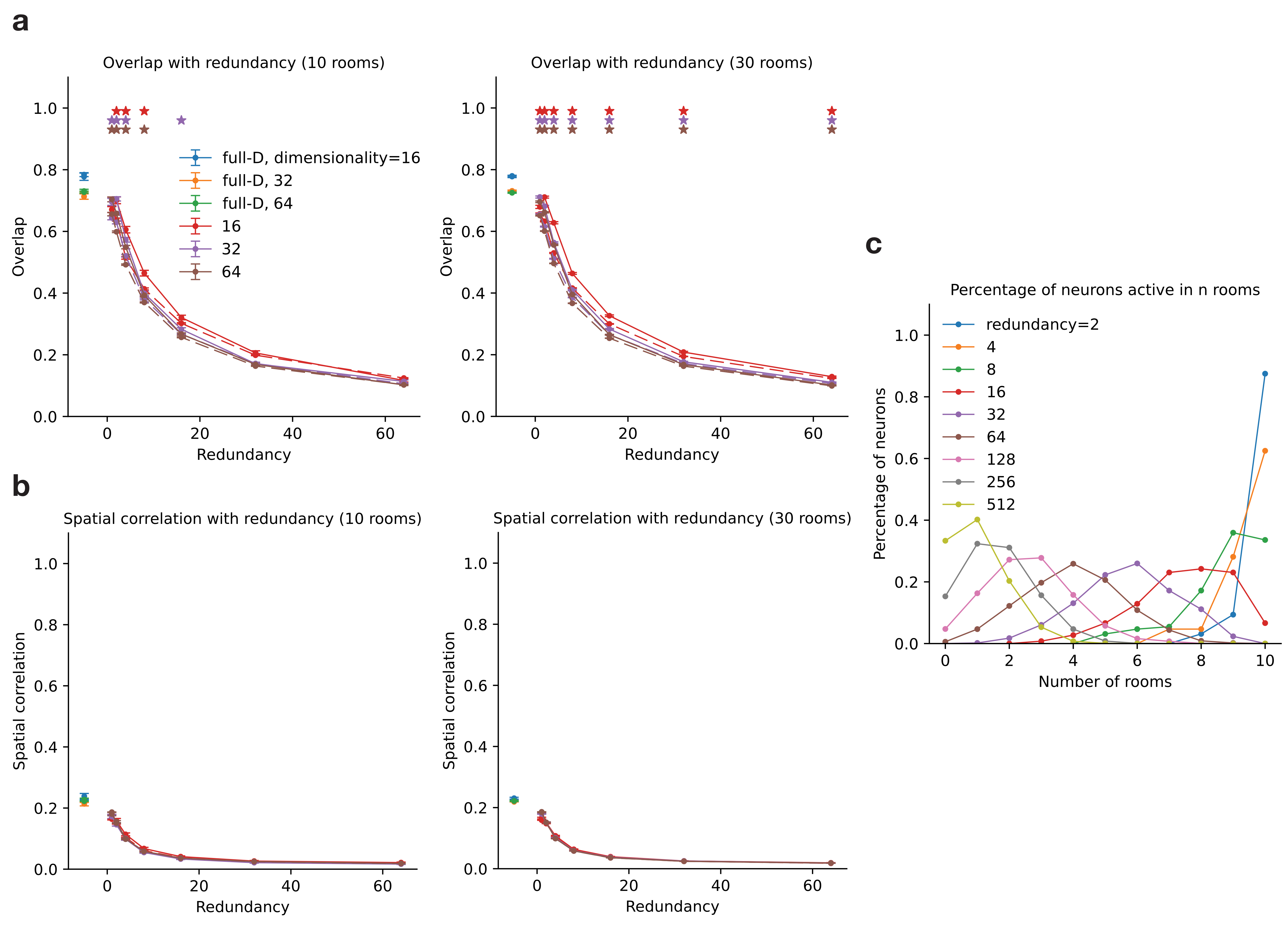

Supplement: S3 Fig — a: Overlap (solid) and shuffle overlap (dashed) between 10 (left) and 30 (right) environments as a function of redundancy (N/Y) for different dimensionality (Y) values. Stars mark where the mean overlap is significantly different from the shuffle mean (t-test, Bonferroni correction with n = 3 for full-D and n = 21 for low-D, see Methods Sect 4). The full-dimensional embedding case (N/Y = 1; Methods Sect 2.1.1; S4 Fig) is plotted at an x-axis value of -1 to differentiate it from the other cases (blue, orange, green). Note the difference significance levels for left versus right by changing the amount of data (10 versus 30 environments). b: Spatial correlation, same as in (a). Note that none of the data is significantly different from random in this case. c: Histogram of percentage of neurons active in n rooms for different redundancies, measured over 10 rooms as in left panels in ( a, b). (TIFF) [file pcbi.1013545.s003.tiff]

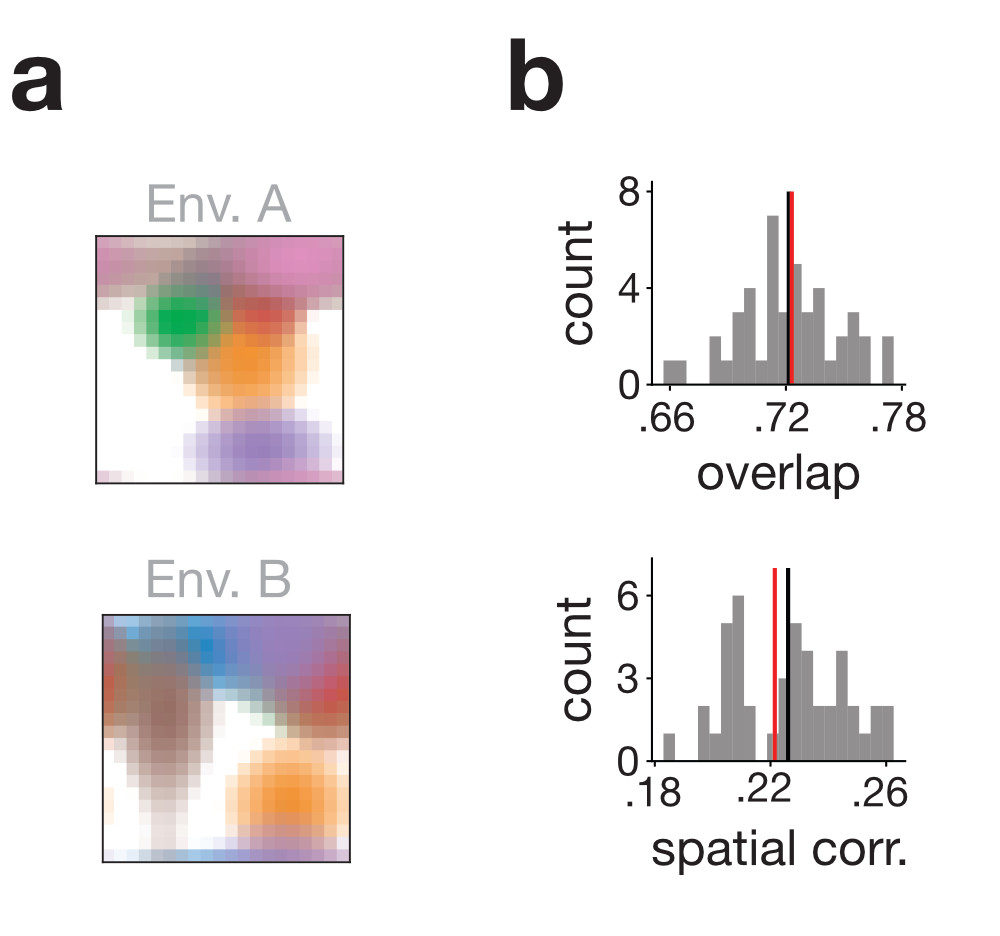

Supplement: S4 Fig — a: Example place field rate maps for two environments. b: Overlap and spatial correlation distributions for 10 rooms, with mean (black) and comparison with a shuffle control (red), showing consistency with truly random remapping. Related to full-D simulations (plotted at x-axis value of -1) from S3a Fig and S3b Fig. (TIFF) [file pcbi.1013545.s004.tiff]

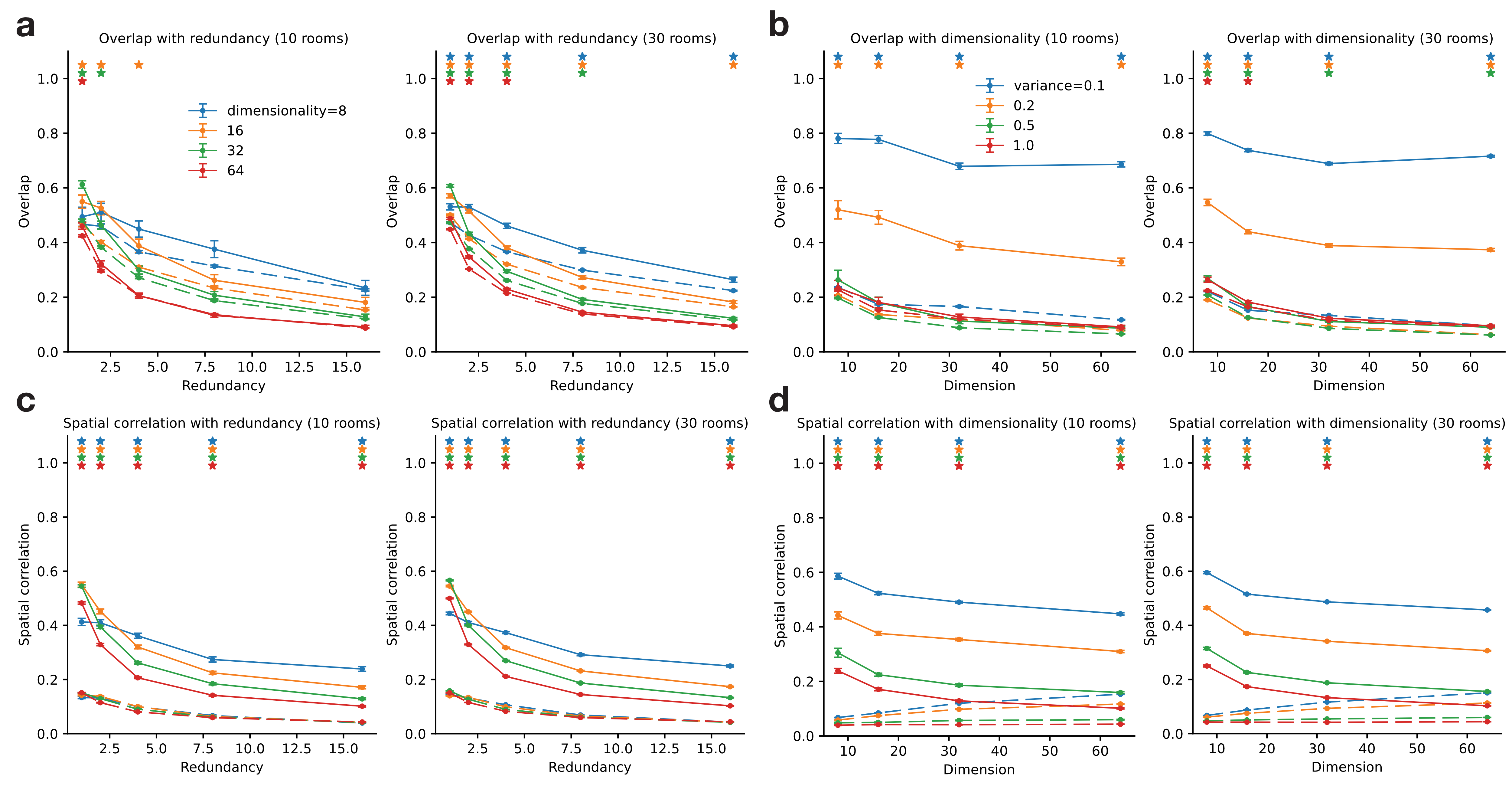

Supplement: S5 Fig — a: Overlap (solid) and shuffle overlap (dashed) between 10 (left) and 30 (right) environments as a function of redundancy (N/Y) for different dimensionality (Y) values. Stars mark where the mean overlap is significantly different from the shuffle mean (t-test, Bonferroni correction with n = 20, see Methods Sect 4). b: Overlap (solid) and shuffle overlap (dashed) between 10 (left) and 30 (right) environments as a function of dimensionality (Y) for different values of cognitive variables variance (σ, see Methods Sect 2.2.1). Stars mark where the mean overlap is significantly different from the shuffle mean (t-test, Bonferroni correction with n = 20, see Methods Sect 4). Note the difference significance levels for left versus right in panels ( a, b) by changing the amount of data (10 versus 30 environments). c: Spatial correlation, same as in (a). d: Spatial correlation same as in (b). (TIFF) [file pcbi.1013545.s005.tiff]

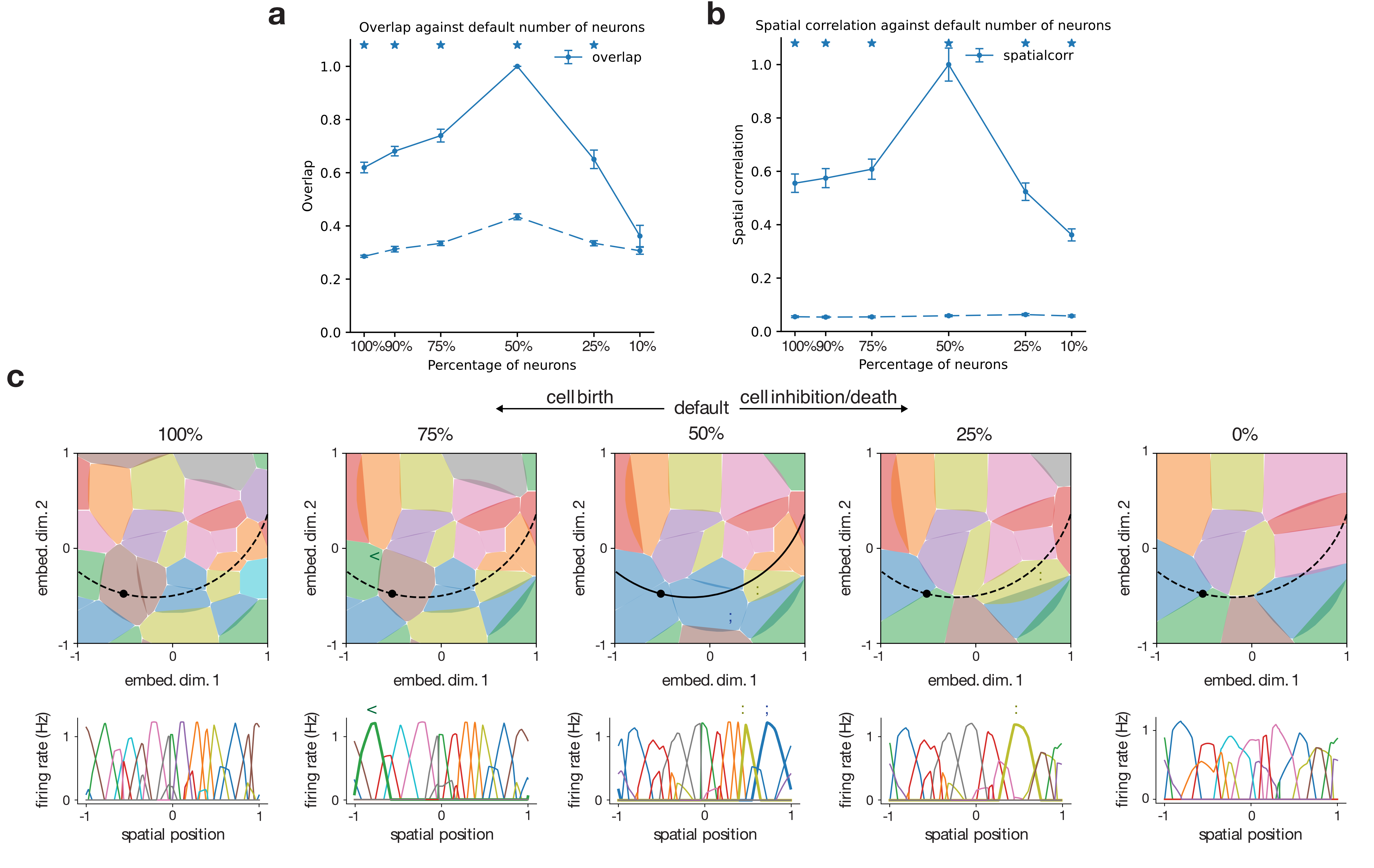

Supplement: S6 Fig — a, b: Overlap and spatial correlation (solid) along with shuffle controls (dashed), comparing “default” network (50% sparsity) to NS remapping where other amounts of sparsity are chosen (spar, see Methods Sect 3.2.2); mean and SEM computed for 5 random selections of suppressed neurons in each case. Stars mark where the mean overlap is significantly different from the shuffle mean (t-test, Bonferroni correction with n = 6, see Methods Sect 4). c: Example trajectories and place fields visualized in angle space (top) and as a function of position (bottom) for different levels of sparsity, following panels ( a, b). Three neurons highlighted (1, 2, & 3) highlighting dropping in and out, and small tuning modulations. For all panels, note that spar>50% indicates cell birth and spar<50% indicates suppression or cell death. (TIFF) [file pcbi.1013545.s006.tiff]

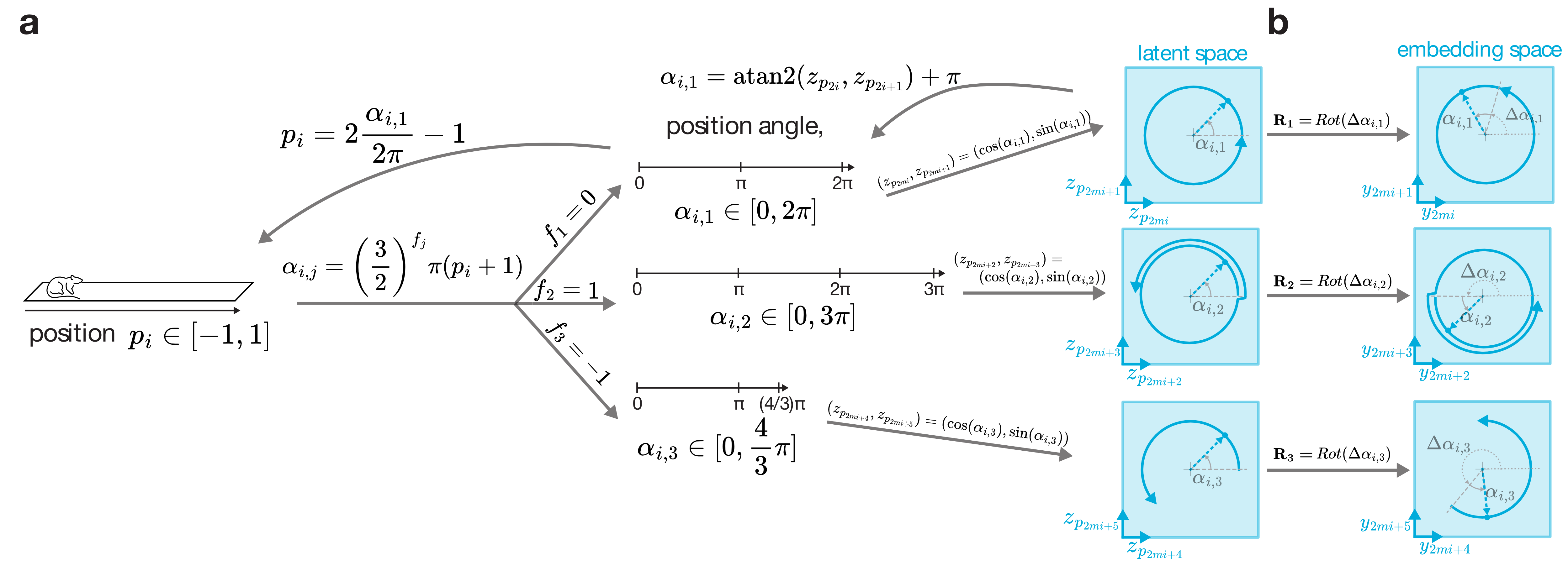

Supplement: S7 Fig — a: Exemplified encoding from a single environmental variable pi∈[−1,1] to its latent representation (zp2mi,zp2m(i+1)−1)∈S1×…×S1 through angular encoding and corresponding decoding. Here using m = 3 modules with frequency parameters f1 = 0, f2 = 1 and f3 = −1. Decoding is done only using the first module due to the restriction f1 = 0. b: Exemplified embedding from this latent representation (zp2mi,zp2m(i+1)−1)∈S1×…×S1 to the embedding space 𝐲∈S1×…×S1 through multiplication with a phase shift matrix 𝐲=𝐑𝐳𝐩 (see Eq 22), constructed with smaller rotation matrices 𝐑1,𝐑2,𝐑3. (TIFF) [file pcbi.1013545.s007.tiff]
