# Supplementary material for: Three types of remapping with linear decoders: A population-geometric perspective
Source: PLoS Comput Biol. 2025 Oct 3;21(10):e1013545. doi: 10.1371/journal.pcbi.1013545 (PMC12510668; doi:10.1371/journal.pcbi.1013545)
Supplement: S1 Table — The notation 2[a,b] stands for all powers of 2j with integers j∈[a,b]. (PDF) [file pcbi.1013545.s008.pdf]

	High dim. (Fig. S3)	Low dim. (Fig. 3e,f, S2a)	Low dim vis. (Fig. 3b-d)	Low dim analysis (Fig. S1, S3)	Grid cells (Fig. 3k,l, S2b)	Grid cells vis. (Fig. 3h-j)
P	2		1	2	2	1
Y	128	128	3	$2^{[4,6]} = 16, 32, 64$	12	4
$\frac{N}{Y}$	1	16	8	$2^{[0,6]} = 1, \ldots, 64$	8	8
N	128	2048	24	$2^{[4,12]} = 16, \ldots, 4096$	96	24
$\mathbf{D}$	$\mathbf{Id}_{N \times N}$	$U_{norm}(Y \times N)$			$U_{norm}(Y \times N)$	
C or M	M	M	M	M	C	C
$\mathbf{z_p}$	$m = 1$				$m = 3$, $f_j \in [0, 1, -1]$	$m = 2$, $f_j \in [0, 1]$
$\mathbf{z}$	$\mathbf{z} = \mathbf{z_p}$				$\mathbf{z} = \mathbf{z_p}$	
$\mathbf{y} = \mathbf{Rz}$	$U_{ortho}(N \times N)$	$U_{ortho}(Y \times N)$			$diag(Rot(2 \times 2))$	
envs	10		2	30	10	2

Table 1: Simulation parameters for encoder-decoder (ED) remapping. The notation $2^{[a,b]}$ stands for all powers of 2^j with integers $j \in [a, b]$
