# Supplementary material for: Three types of remapping with linear decoders: A population-geometric perspective
Source: PLoS Comput Biol. 2025 Oct 3;21(10):e1013545. doi: 10.1371/journal.pcbi.1013545 (PMC12510668; doi:10.1371/journal.pcbi.1013545)
Supplement: S2 Table — The notation 2[a,b] stands for all powers of 2j with integers j∈[a,b]. (PDF) [file pcbi.1013545.s009.pdf]

<table>
<tr><th></th><th>Space-feature (Fig. 4e,f, S2c)</th><th>Space-feature vis. (Fig. 4b-d)</th><th>Space-feature analysis (Fig. S5)</th><th>Reward vis. (Fig. 6c-f)</th><th>Implicit-space (Fig. 4k,l, S2d)</th><th>Implicit-space vis. (Fig. 4h-j)</th></tr>
<tr><td>P</td><td>2</td><td>1</td><td>2</td><td>1</td><td>2</td><td>1</td></tr>
<tr><td>Y</td><td>64</td><td>4</td><td>$2^{[3,6]} = 8, \ldots, 64$</td><td>4</td><td>64</td><td>4</td></tr>
<tr><td>$\frac{N}{Y}$</td><td>16</td><td>8</td><td>$2^{[0,4]} = 1, \ldots, 16$</td><td>4</td><td>16</td><td>8</td></tr>
<tr><td>N</td><td>1024</td><td>32</td><td>$2^{[3,10]} = 8, \ldots, 1024$</td><td>16</td><td>1024</td><td>32</td></tr>
<tr><td>$\mathbf{D}$</td><td colspan="4">$U_{norm}(Y \times N)$</td><td colspan="2">$U_{norm}(Y \times N)$</td></tr>
<tr><td>C or M</td><td colspan="3">CM</td><td>CM and pM</td><td colspan="2">M</td></tr>
<tr><td>$\mathbf{c}$</td><td colspan="2">$U([-1,1]^C) + GP(\mathbf{0}, K)$</td><td>$\mathcal{N}(\mathbf{0}, \sigma) + GP(\mathbf{0}, \sigma K)$, σ=0.1,0.2,0.5,1</td><td>$\text{pdf}_{\mathcal{N}(\mu_r, \sigma Id)}$, $\sigma = 0.01$</td><td colspan="2">$U([-1,1]^C) + GP(\mathbf{0}, K)$</td></tr>
<tr><td>$\mathbf{z}$</td><td colspan="4">$\mathbf{z} = (\mathbf{z_p}, \mathbf{z_c})$</td><td colspan="2">$\mathbf{z} = \mathbf{z_c}$</td></tr>
<tr><td>$\mathbf{y}$</td><td colspan="4">$\mathbf{y} = \mathbf{z}$, $\|\mathbf{y}_{j \in P}\| = \|\mathbf{y}_{j \in C}\| = 1/\sqrt{2}$</td><td colspan="2">$\mathbf{y} = \mathbf{z}$</td></tr>
<tr><td>envs</td><td>10</td><td>3</td><td>30</td><td>2</td><td>10</td><td>3</td></tr>
</table>

Table 2: Simulation parameters for mixed-selective (MS) remapping. The notation $2^{[a,b]}$ stands for all powers of 2^j with integers $j \in [a, b]$
