# Supplementary material for: Three types of remapping with linear decoders: A population-geometric perspective
Source: PLoS Comput Biol. 2025 Oct 3;21(10):e1013545. doi: 10.1371/journal.pcbi.1013545 (PMC12510668; doi:10.1371/journal.pcbi.1013545)
Supplement: S3 Table — The notation 2[a,b] stands for all powers of 2j with integers j∈[a,b]. (PDF) [file pcbi.1013545.s010.pdf]

	Null-space (Fig. S2e)	Null-space vis. (Fig. 5e-g)	Null-space analysis (Fig. S6)
P	2	1	2
Y	128	3	16
$\frac{N}{Y}$	32	16	16
N	4096	48	256
$\mathbf{D}$	$U_{norm}(Y \times N)$		
C or M	M		
$spar$	1		[1, 0.9, 0.75, 0.5, 0.25, 0.1]
$\mathbf{T}$	$\mathbf{T}_{active} = 10.5$		
$\mathbf{z}$	$\mathbf{z} = \mathbf{z_p}$		
$\mathbf{y} = \mathbf{Rz}$	$U_{ortho}(Y \times N)$		
envs	1	1	5

Table 3: Simulation parameters for null-space (NS) remapping.
