# Supplementary material for: Three types of remapping with linear decoders: A population-geometric perspective
Source: PLoS Comput Biol. 2025 Oct 3;21(10):e1013545. doi: 10.1371/journal.pcbi.1013545 (PMC12510668; doi:10.1371/journal.pcbi.1013545)
Supplement: Attachment — Submitted filename: PCB Remapping Rebuttal (1).pdf [file pcbi.1013545.s011.pdf]

Response to reviewers

Summary of significant changes made

We would like to thank the two reviewers for the time and effort spent on the evaluation of our manuscript. We have revised the manuscript in accordance with the points brought up in the reviewer comments, and we feel that the new version is substantially more rigorous and clearer in explanation. We summarize four main points of modification here:

1. We now include **systematic parameter sweeps** for each remapping type, enabling a more thorough understanding of how model parameters affect remapping statistics, as well as demonstrating that the conclusions drawn are robust and not fine-tuned. This consists of:
    - Hyperparameter sweeps (Figure S1): demonstrates how latent dimensionality and redundancy (ratio of network size to dimensionality) affect place cell statistics (mean place field size and the percentage of active cells per environment).
    - Encoder-decoder remapping (Figure S3): parameter sweeps through dimensionality and redundancy across sets of environments.
    - Mixed-selective remapping (Figure S5): parameter sweeps through dimensionality and redundancy, as well as variability in cognitive variable trajectories.
    - Null-space remapping (New Figure 5 & Figure S6): parameter sweep through number of silenced or activated neurons, along with new quantifications.

    With these parameter sweeps, our theory is now much more thoroughly explored and the results that we highlight can be better treated as representative examples of each case. To incorporate these new results into the main text, we have added **substantial revisions to the text** for the three types of remapping **in the second half of the Results**.

2. The Discussion now includes a new **summary table** (Table 1), which highlights the identifying signatures of each remapping type, as well as their **connections to an extensive list of experimental and computational studies** (with more details given in Methods Section 5). This table now directly links our work to the experimental literature, thereby demonstrating its relevance for the experimental study of remapping.

3. We have made extensive revisions to the text, including improved descriptions in the Results, as well as an **extensively revised Methods section**, complete with high-level explanations of each major part of the theory and simulations.

4. We expand on and clarify the use of **angular coding** for the internal representation and its relationship to periodic spatial environments and periodic activity. Specifically, we now further highlight that the angular code is not necessary for the theoretical characterization of three types of remapping, which is compatible with any one-to-one mapping from position to latent variables (Results lines 107-110, 201-208; Discussion lines 397-404). Second, we add further motivation in the Methods (lines 520-533) and clarify that a periodic or angular variable representation does not imply periodic neural activity (lines 544-555). We thus

contend that our use of circular trajectories for spatial position found in Fig. 3a-f, Fig. 4a-f, and Fig. 5 is a useful abstraction. It enables us to study remapping with the minimal set of latent variables, and we stress that higher-dimensional settings will virtually never exhibit periodic neural activity.

Below you will find a point-by-point response to each of the reviewer comments, with the original reviewer comments in **gray**, and our response in **black**.

Yours sincerely,
Guillermo Martín-Sánchez, Christian K. Machens, & William F. Podlaski

---

Reviewer 1

This manuscript introduces a novel theoretical framework to understand hippocampal remapping through a population geometry and neural coding perspective. The authors propose three distinct mechanisms of remapping based on linearly-decodable latent space representations: encoder-decoder (ED) remapping, mixed-selective (MS) remapping, and nullspace (NS) remapping. The framework offers a unifying approach to interpret the diverse experimental observations and theoretical models in the field.
The work is significant as it attempts to bridge different perspectives on hippocampal function and provides a systematic way to categorize various remapping phenomena. The mathematical formalism is valuable and could potentially be applied beyond the hippocampus to understand neural variability more broadly.

We thank the reviewer for their encouraging words.

My only major concern is that the methods section is poorly structured and was hard to read, please:
- Add conceptual overview subsections before mathematical details
- Create a glossary of symbols
- Add schematic diagrams of the mathematical constructs
- Refer to specific subsections of the methods in the results section so you do not have to read through everything to find what you are looking for.
- Although I liked the first part of the results, including Fig 1, consider including more of the methods before the main results, particularly how the results were obtained (optimization, cost, etc)

We have now significantly expanded and clarified many sections of the Methods, and we hope our methodology and explanations are much clearer. Specifically, we made several changes according to the reviewer's above suggestions.

In the Methods themselves, we did the following:

- We added a glossary table of symbols to the beginning of the methods (Table 2).
- We added extensive conceptual overviews in plain text to several parts of the methods, notably in sections 1.1, 1.2, and 1.3 (lines 457-555).
- We added schematic equations (e.g., Eqs. (6) and (14)) and an additional supplementary figure for grid realignment (Fig. S7) for improved clarity of our theoretical framework and methodology.

Furthermore, in the main text we did the following:

- We improved cross-referencing of parts of the Methods in the main text and now refer to specific Methods sections (e.g., lines 101-102, 110, 115-116, 121, etc.)
- We expanded the description of the RNN simulations in the Results (see subsection "Nonlinear encoding and linear decoding in an autoencoder network", lines 144-160).

Other than this, the manuscript could be published as is, but I include some additional concerns that I believe would increase the quality and can be taken as suggestions rather than requests.

Experimental validation and predictions:
The manuscript lacks direct validation against experimental data. While the authors discuss how their framework relates to existing experimental findings, they do not test their model predictions against specific datasets. This limits the immediate impact of the work.

Suggestion: Incorporate analyses of published datasets that demonstrate how the proposed remapping mechanisms explain observed neural activity patterns.

We agree with the reviewer that a more direct relation between our theoretical framework and experimental data would strengthen its impact. In order to address this at a high level, as well as to better explain how the different remapping types can be distinguished, we have added a table to the main text summarizing the main results (Table 1). In this table, we also list and partition a large number of experimental studies on remapping into the different types based on the results that they demonstrate. We feel that this addition has significantly improved the clarity and takeaways of our work as well as its connection to experimental studies on remapping, and we thank the reviewer for this suggestion.

For example, how does the distribution of field sizes compare to experimental data?

We now include an additional supplementary figure (Figure S1) which studies how particular hyperparameters (dimensionality and redundancy, ratio of network size to dimensionality) affect place field size and percentage of active cells per environment. As shown in the figure, our framework is compatible with a wide range of place field sizes. As it stands, we could therefore tune these hyperparameters to match any particular experimental datasets. However, our framework provides no specific guidelines or normative accounts for particular parameter choices. Given our focus on population-level statistics of remapping across environments, we felt that a more detailed description of these single-neuron statistics was out of the scope of this

work. Interestingly, however, a recent study (Mainali et al. 2025, ref [64]) modeled place field statistics from data using thresholded Gaussian process inputs similar to what we do here for mixed-selective remapping. We suspect that our model would also be capable of matching such distributions from real neural data.

How does the correlation structure between environments change as a function of noise amplitude?

We are unsure which “noise” the reviewer is referring to, but we take the opportunity to point out a few characteristics of our model and results that we hope will address this question. First, we note that our theoretical framework assumes deterministic rate maps r(p) across position in each environment, which is also the case in our simulations. Thus, the only notion of noise that we address in our results pertains to the variance of trajectories across environments for each of the remapping types. With our parameter sweeps, we now directly explore and demonstrate this, with variance in ED remapping corresponding to the embedding space dimensionality (Figure S3), variance in MS remapping corresponding to dimensionality and redundancy of the latent space as well as the explicit variance of the Gaussian process (Figure S5), and variance in NS remapping corresponding to the sparsity or density of the modulation (Figure S6).

Compare the predictions of encoder-decoder remapping with changes in environment geometry such as in Krupic et al 2015, which also reminds me that the grid cells used for grid alignment seems square, it would be nice to plot these grid cells to show their tuning.

We find the relationship between environmental geometry and cell tuning to be extremely interesting, yet we argue that such a question is out of the scope of our work. Given the representational nature of our framework, environmental geometry could be incorporated into the spatial input of the model. If we take seriously the notion that environmental geometry affects the metric of space in the model, then this could be incorporated into the encoding of the environmental variables into latent space, which we now briefly mention in the Methods (lines 530-533). Alternatively, spatial input features based on boundary vector cells or similar representations could provide a natural way of explicitly modeling environment changes, which we also now mention in the Discussion (line 380). These modifications serve as particular examples that could be explored in future work.

Next, the reviewer is correct in noting that the grid-like input we provide to our model in Fig. 3g-I is indeed reflective of a square grid. We made this choice for simplicity, but a twisted torus topology would instead result in a hexagonal lattice. We have made a note of this in the Methods (lines 609-611). We do not plot these grid inputs directly, but we now include a supplementary figure that better explains the grid realignment model (Figure S7).

The necessity of angular coding for generating place-like fields could be expanded.

We thank the reviewer for bringing up this point, as it was not sufficiently well explained in the original submission. As mentioned above in the summary, we have now made several changes

to the text to highlight that the angular code is not necessary for our theory (Results lines 107-110, 201-208; Discussion lines 397-404). We have also added additional motivation and justification in the Methods which we include here:

Methods lines 522-530:

> *There are two main reasons that motivate a more nonlinear, curved mapping. First, neural codes should be energy efficient, and are often modeled with explicit or implicit activity regularization or normalization which confines activity within or onto a hypersphere around the origin [128, 129]. From this perspective, it is the direction rather than the magnitude that determines the representation. Second, place field activity is often confined to one or multiple localized areas of space. This motivates a curved trajectory that only locally aligns with each neuron's preferred tuning vector, which in ideal abstraction becomes a periodic or toroidal map [130]. In contrast, a purely linear representation is only consistent with monotonic tuning curves. Therefore, we choose an angular code as a simplistic model that accounts for both energy-efficient coding and localized place-field-like tuning.*

Methods lines 545-551:

> *We stress that in the more general case of a multi-dimensional latent variable representation, periodic codes do not necessarily mean that neural activity will be periodic — this will only come about in the special case that all latent variables have either the same period, or periods that divide evenly into one another. Indeed, this is precisely why periodic grid cell input can account for localized, non-periodic place cell activity [60,61], as we also see in the grid realignment ED remapping model (Fig. 3g-l), though the approximate periodicity induced can account for place cells with multiple fields in large environments [27,28]. The addition of non-periodic cognitive variables will further diversify activity (Fig. 4).*

We hope that these additional points sufficiently clarify the role of angular coding in our framework.

Nullspace remapping:
The nullspace remapping mechanism is the most novel contribution but could get more attention. The authors do not fully explore the implications of this mechanism, particularly:

- How does the nullspace component depend on the choice of encoder/decoder pairs?

In principle, the nullspace component fully depends on the choice of encoder/decoder pairs — when the encoding changes, the nullspace component must also "reorient" in neural state space in order to remain in the nullspace of the latent space. As we had demonstrated in Fig. S2 (formerly Fig. S1), changes to encoders and decoders in ED remapping results in substantial changes to the nullspace.

- What transformations of the nullspace function $v(\cdot)$ would leave the decoding invariant?

Importantly, by definition, all possible nullspace functions leave the decoding invariant, as they are in the null space of the decoder.

- What constraints on the nullspace would be most biologically plausible?

The constraints on the nullspace depends on the specific network architecture employed. Our RNN model can be phrased as solving a constrained optimization problem: to optimally represent the latent variables (spatial and cognitive) such that they can be read out with a linear decoder, using non-negative ($r>0$) and sparse activities (Eqs. (3) & (33)). In our formulation, the amount of sparsity is directly linked to the thresholds (or excitabilities) of the neurons. NS remapping acts on these threshold parameters, thereby modulating the excitability of the neurons, and the sparsity of the population representation Additional constraints could likely be included, but we think that a minimum amount of constraints best serves the conceptual nature of our paper.

To make things more concrete, we now include further discussion of how the RNN model we employ affects the null space component as well as null-space remapping (lines 745-761).

Suggestion: Expand the analysis of nullspace remapping, perhaps including a case where v is restricted to be linear, which would build intuition while maintaining analytical tractability. Investigate how nullspace remapping would manifest if E were trained through learning rather than fixed.

Following this suggestion as well as those of Reviewer 2, we decided to study nullspace remapping more in depth, by choosing different numbers of neurons to either add (cell birth) or silence (cell suppression/death) and comparing the resulting place code (Fig. 5 & S6). We feel that this has significantly expanded the experimental relevance of nullspace remapping. As for the particular suggestions of the reviewer to study a linear nullspace term, or the case in which E is trained — we find these suggestions to be interesting for future work, but we feel that they are out of the scope of this current study. The nullspace term is perhaps the most elusive part of our framework, which we make concrete by choosing a particular neural network model. However, there is much more to explore in the future about the implications of this term!

The trial-to-trial variability described in the nullspace remapping mechanism seems to create a potential inconsistency with experimental observations.
If nullspace remapping were the only mechanism at play, would it not suggest that animals would show remapping on every trial, regardless of familiarity or novelty? If so, this contradicts well-established experimental findings where Place cells show stable spatial firing fields across multiple trials in familiar environments, global remapping is typically observed when environments change significantly, and stability of place cell representations increases with familiarity. This would be worth discussing.

This is an interesting and important point that we are happy to discuss and clarify. As we note in the Results and Methods (lines 162-167 & 493-511) we utilize a broad definition of remapping

that includes any changes in firing rates for the same position, no matter how subtle. Thus, small variations in activity on a trial-to-trial basis do count as minimal "remapping" from our definition. Importantly, this does not mean that the place code is not stable – instead, trial-to-trial variability would manifest as small amounts of "local rate remapping" that would yield stable place fields on average. Now, if such variability were systematic and drifting over time, then it would result in representational drift, as we also now mention in the Discussion and Methods (lines 386-387, 417-422; lines 847-862).

In other words, in our formalism, trial-to-trial variability could indeed be seen as a form of null-space "remapping" which is fully consistent with stable rate maps. This could be an interesting account of the commonly-observed variability seen in place cells (e.g., Fenton & Muller 1998, ref [99]).

Reviewer 2

In this work, the authors provide a geometrical interpretation of remapping, a widely observed phenomenon where place cells change their firing properties (such as firing rate or preferred firing location) in response to contextual changes. They propose a population-level view of position coding as a trajectory in a low-dimensional latent space plus an off-manifold, non-linear component, and then use this model to define three possible mechanisms that could underlie remapping at the population level.

The effort to synthesize existing models of remapping (such as attractor maps or latent space shifts) into a unified, population-centric framework is appreciated and relevant to the hippocampal community. The paper is clearly written, and the figures are well-structured. It is evident that the authors made an effort to guide the reader through their framework using clear illustrations. However, in its current form, the paper neither provides a comprehensive theoretical account nor yields clear insights into remapping mechanisms observed in real data. Below are a few comments that I recommend addressing before the manuscript can be considered for publication.

We thank the reviewer for their kind words and constructive criticism of our work.

* If the authors aim to present a theoretical framework, then a more thorough exploration of the phenomenology is needed. Specifically, which observable features serve as clear fingerprints of each remapping type, and which are merely consequences of modeling choices (such as the number of neurons, RNN implementation, angular latent space, etc.)? For example, the first (encoder-decoder) and second (mixed-selectivity) remapping types appear to produce different outcomes in terms of spatial overlap and correlations, but this is illustrated only with a single underwhelming simulation instead of investigated on a systematic level. The framework could be leveraged to extract heuristics that distinguish remapping types based on population-level

properties (e.g. tuning overlap and correlation), offering testable predictions for future work and experimental validation.

We appreciate the reviewer's suggestion and agree that a more thorough study of our framework was merited. To address this, we now include extensive parameter sweeps in order to explore the diversity of characteristics that each remapping type expresses, as well as to determine the robustness of the claims that we make (Figs. S1, S3, S5, S6). Furthermore, we provide a summary table (Table 1, Methods Section 5) at the end of the paper which, as much as is possible, establishes clear signatures of each type as the reviewer suggests. Specifically, we did the following:

- Hyperparameter sweeps (Figure S1): demonstration of how latent dimensionality and redundancy (network size divided by latent dimensionality) affect place cell statistics (mean place field size and the percentage of active cells per environment). Result: the autoencoder model class is capable of generating a wide array of statistics, primarily controlled by redundancy, which affect the raw values of overlap and spatial correlation (but not necessarily their randomness).
- Encoder-decoder remapping (Figure S3): parameter sweeps through dimensionality and redundancy across sets of 10 and 30 distinct environments.
  Signature: all ED models exhibit random spatial correlation across environments; most models have low-dimensional population structure resulting in non-random overlap, which may appear "seemingly random" in networks with high redundancy; the exception to this is the full-D multi-chart model which is the only model with truly random overlap, because it lacks any shared population structure across environments (also see Fig. S4).
- Mixed-selective remapping (Figure S5): parameter sweeps through dimensionality and redundancy across sets of 10 and 30 distinct environments, followed by sweeps through variability in cognitive variable trajectories (defined by Gaussian Process variance).
  Signature: most MS models are characterized by non-random overlap and non-random spatial correlation (i.e., partial and rate remapping); this again may appear "seemingly random" with high redundancy and variance in the trajectories; the exception to these signatures is implicit-space MS, which can approximate random spatial correlation if the trajectories are made variable enough.
- Null-space remapping (New Figure 5 & Figure S6): parameter sweep through number of silenced or activated neurons, along with quantification via overlap and spatial correlation measures. Expands the notion of nullspace remapping to changes in excitability, cell birth/death ("participation modulation") and experimental suppression.
  Signature: NS remapping nearly always exhibits gradual activity changes with clearly non-random overlap and non-random spatial correlation similar to MS remapping; in the extreme case of dense modulation, it may exhibit "seemingly random" overlap.

With these parameter sweeps and updates, our theory is now much more thoroughly explored and the results that we highlight can be better treated as representative examples of each case. This helps our work to serve as a more robust and well-understood theoretical framework of remapping.

* Relatedly, the lack of any application to real data limits the scope of the paper. While a computational paper can stand on its own - and I believe this paper could very well do so if the previous point is addressed - the framework would be significantly strengthened by showing how these remapping fingerprints appear (or fail to appear) in experimental datasets. There is a wealth of open-access hippocampal recordings that could be used for this purpose.

We agree with the reviewer that our original manuscript lacked experimental context, and we now have added explicit reference to experimental studies where each remapping type is identified. Specifically, in Table 1, we now list the fingerprints of each remapping type, alongside a long list of experimental papers partitioned into the type of remapping that they are consistent with (also see Methods Section 5).

That said, we argue that a more explicit comparison between our framework and experimental data is out of the scope of this current work. First and foremost, this is because the main aim of our paper was to present a general theory of remapping rather than focusing on specific experimental datasets. Furthermore, while more direct comparisons with data would be interesting to explore in future work, our results suggest that it would be difficult and would likely require its own comprehensive and standalone study. This includes the fact that several of the models that we study have overlapping features, and may require extensive amounts of data (e.g., place cell activity in >10 environments) to identify population structure (e.g., the “seemingly random” cases). Thus, while we could tune the hyperparameters of the model to fit the statistics of a particular dataset, drawing clear conclusions from it would likely require a directed experimental question which is out of the scope of this more theoretically-oriented paper.

* I also find the beginning of the Results section unnecessarily dense. The initial decoding-based formulation feels superfluous, especially since the encoding model $r=Ez+v$ already contains all the necessary components to introduce and distinguish the three remapping types. The encoding view is not only more intuitive from a neuroscience perspective but also more directly tied to how remapping is typically conceptualized. Starting from encoding would make the paper much easier to follow and more logically structured.

We agree with the reviewer that the encoder perspective is crucial for understanding the three types of remapping. We have now rewritten this section in order to more directly introduce the pseudo-linear encoder as early as possible (lines 112-123). We now explain that the linear decoder is simply the fundamental assumption of our model, whose key purpose is to motivate the pseudo-linear encoder (see additional details and motivation in the Methods lines 473-491). We hope this streamlining helps to improve the clarity of the theoretical approach.

* The choice to model latent position as an angular (periodic) variable is also worth revisiting. This might be reasonable for certain experimental settings (such as circular mazes or virtual environments with repeating structure), but it seems problematic to assume that all spatial coding relies on periodic representations. Is this assumption required to produce place fields or

to differentiate remapping types? If so, the implications should be discussed. If not, it would be helpful to show that the results hold in a non-periodic latent space as well.

We thank the reviewer for bringing up this point, as it was not sufficiently well explained in the original submission. As mentioned above in the summary and in our response to Reviewer 1, we have now made several changes to the text to highlight that the angular code is not necessary for our theory (107-110, 201-208; Discussion lines 397-404). We have also added additional motivation and justification in the Methods which we include here:

Methods lines 522-530:

> *There are two main reasons that motivate a more nonlinear, curved mapping. First, neural codes should be energy efficient, and are often modeled with explicit or implicit activity regularization or normalization which confines activity within or onto a hypersphere around the origin [128, 129]. From this perspective, it is the direction rather than the magnitude that determines the representation. Second, place field activity is often confined to one or multiple localized areas of space. This motivates a curved trajectory that only locally aligns with each neuron's preferred tuning vector, which in ideal abstraction becomes a periodic or toroidal map [130]. In contrast, a purely linear representation is only consistent with monotonic tuning curves. Therefore, we choose an angular code as a simplistic model that accounts for both energy-efficient coding and localized place-field-like tuning.*

Methods lines 545-551:

> *We stress that in the more general case of a multi-dimensional latent variable representation, periodic codes do not necessarily mean that neural activity will be periodic — this will only come about in the special case that all latent variables have either the same period, or periods that divide evenly into one another. Indeed, this is precisely why periodic grid cell input can account for localized, non-periodic place cell activity [60,61], as we also see in the grid realignment ED remapping model (Fig. 3g-l), though the approximate periodicity induced can account for place cells with multiple fields in large environments [27,28]. The addition of non-periodic cognitive variables will further diversify activity (Fig. 4).*

We hope that these additional points sufficiently clarify the role of angular coding in our framework.

* Finally, I am not sure what is gained from the set of single-example simulations (e.g., cell silencing, reward modulation, etc.). These feel disconnected from the core framework and do not offer clear conceptual insight. Rather than testing a large number of scenarios each loosely mapped to a different experiment, the paper would benefit more from a focused and systematic analysis of the population-level consequences of each remapping strategy (see my first point). Such an analysis would help ground the framework in observable phenomena and make it more useful to the community.

The point of the simulations was to highlight the experimental relevance of the three remapping types. We agree that they may have appeared as single examples whose generality seemed

doubtful. To counter this impression and clarify this section, we have now taken up the suggestion of the reviewer and included systematic parameter sweeps for all simulated models. With these parameter sweeps, we now show that the single-example simulations for ED, MS, and NS remapping indeed can serve as representative examples of each remapping type.

As for the specific cell silencing and reward modulation examples, we have included those as demonstrations of how our framework might be adapted to particular experimental cases. We have rewritten these sections to clarify their purpose and their link to the experimental literature.

In addition, we summarize all results in a new summary table, thereby clarifying the implications of our framework and strengthening its connections to the experimental literature.

Overall, the paper presents an interesting perspective and an appealing geometric formulation. However, I believe that with a more systematic exploration of the core model, a clearer presentation, and at least a minimal link to data, the paper could make a much stronger and more lasting contribution.

The revised version now includes a more systematic exploration of the core model, we have rewritten large parts of the text to make the presentation clearer, and we have provided a better link to experimental remapping studies. We believe these changes have made the paper much better, and we thank the reviewer for their insightful feedback and fair assessment of our work.
